# Optimising the European Code Against Cancer, 5th edition, to increase awareness of avoidable cancer risks in all socioeconomic groups

**DOI:** 10.1002/1878-0261.70196

**Published:** 2026-01-16

**Authors:** Eleni Mantzari, Katherine Brain, Kathelijne M. H. H. Bessems, Janne Bigaard, Liacine Bouaoun, Erica D'Souza, Carolina Espina, Cristiana Fonseca, Ariadna Feliu, Dafina Petrova, David Ritchie, Joachim Schüz, Violaine Roggeri, Hajo Zeeb, Theresa M. Marteau

**Affiliations:** ^1^ Behaviour and Health Research Unit University of Cambridge UK; ^2^ Division of Population Medicine, School of Medicine Cardiff University UK; ^3^ NUTRIM Institute of Nutrition and Translational Research in Metabolism, Department of Health Promotion, Faculty of Health Maastricht University The Netherlands; ^4^ Prevention & Information Danish Cancer Society Copenhagen Denmark; ^5^ Environment and Lifestyle Epidemiology Branch, International Agency for Research on Cancer (IARC‐WHO) Lyon France; ^6^ Health Education Department, Portuguese League Against Cancer – Northern Branch (LPCC – NRN) Porto Portugal; ^7^ Instituto de Investigación Biosanitaria ibs.GRANADA Spain; ^8^ Escuela Andaluza de Salud Pública Granada Spain; ^9^ CIBER de Epidemiología y Salud Pública (CIBERESP) Madrid Spain; ^10^ Medical Oncology, Hospital Universitario Virgen de las Nieves Granada Spain; ^11^ European Agency for Safety and Health at Work Bilbao Spain; ^12^ Leibniz‐Institute for Prevention Research and Epidemiology – BIPS Bremen Germany

**Keywords:** cancer, cancer prevention, cancer risk factors, European Code Against Cancer, risk communication, risk messages

## Abstract

Although cancer is a leading cause of death in the European Union, around 40% of cases are preventable. The European Code Against Cancer (ECAC) was developed to inform citizens about key cancer‐risk‐reducing actions. This study aimed to identify effective ways to present the 5th edition of the code (ECAC5) to optimise awareness of cancer risks in all socioeconomic groups. Using a 2 × 3 × 2 factorial design, 10 027 participants from eight countries were randomised online to receive ‘no message’ or one of 10 ECAC5 formats differing in message content (cancer risks: present/absent), length of message on cancer prevention actions (longer/shorter/absent) or format (text‐only/text with images). The primary outcome was awareness of 16 avoidable cancer risks. Overall mean number of risks recalled was 2.40 (standard deviation: 1.72; range 0–14). Recall was highest when messages included risk information. Adding prevention messages to risk information did not improve risk factor recall. Message length and images had no significant impact. Effects were similar across levels of education and countries. Combined information about risk factors and preventive actions has the potential to equitably increase citizens’ very low cancer prevention awareness. How this awareness might change over time or lead to behaviour change is unknown and should be the focus of future evaluations.

## Graphical abstract

The 5th edition of the European Code Against Cancer (ECAC5) includes additional information on the avoidable risk factors for cancer, as well as the actions citizens can take to lower their cancer risk. Based on our research results, we conclude that including explicit information about the avoidable risk factors for cancer in the ECAC5 increases the very low awareness of cancer risks among EU citizens.

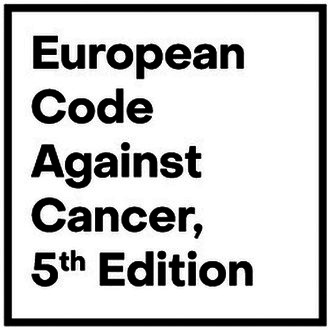

**Abbreviations**
ECAC: European Code Against Cancer
EU: European Union
IARC: International Agency for Research on Cancer
WHO: World Health Organization

## 1 Introduction

Cancer is a major contributor to the global burden of disease, being a significant cause of worldwide morbidity [1, 2] and the second leading cause of death globally [3, 4]. The International Agency for Research on Cancer (IARC), a specialised cancer agency of the World Health Organization (WHO), estimates that between 30% and 50% of all cases of cancer are preventable [5, 6]. The leading risk factors contributing to the global cancer burden, including early‐onset cancers, are behavioural. These include tobacco use, alcohol consumption and dietary behaviours linked to overweight and obesity [7]. Individuals from lower socioeconomic groups are at disproportionately higher risk of cancer incidence and mortality [8, 9, 10, 11, 12], reflecting the social patterning of these behavioural risk factors, which are more prevalent in socioeconomically disadvantaged groups [13, 14, 15].

Accordingly, a substantial proportion of the global cancer burden could be prevented by changing behaviours linked to cancer development. Changing behaviour, however, is difficult and requires multiple interventions at scale across populations [16]. One commonly used approach is to inform people of these risks, in an attempt to motivate them to change their behaviour. Providing information, however, has—at best—a small effect on behaviour [17, 18]. Nonetheless, citizens have a right to reliable information that may inform their choices. This is especially important, given that a large proportion of the population lacks awareness of some of the leading avoidable cancer risk factors when asked to freely recall them. Although low levels of awareness of cancer risks are complex, influenced by multiple individual, social and contextual factors [19, 20, 21, 22], of which lack of information is only one, providing reliable information to those who lack it is arguably a necessary prerequisite to increasing awareness. Low levels of awareness are particularly marked in those in the most socioeconomically deprived groups [e.g. 23]. For example, in an unpublished study of cancer risk awareness in the UK population, 43% of those in the highest socioeconomic group and 27% in the lowest socioeconomic group spontaneously mentioned alcohol as a cancer risk factor [23]. This mirrors findings from other countries in which only a small minority of citizens name alcohol as a risk factor for cancer [24, 25, 26]. Providing information on cancer risk factors has the potential not only to increase awareness but also to increase public support for effective interventions which most often require regulation or legislation. Citizens may be more receptive to policy makers' decisions involving restriction on availability and marketing, warning labels and fiscal policies, if they understand the rationale for those policies [e.g. 27, 28, 29].

The European Commission has funded the development of the European Code Against Cancer (ECAC) to inform citizens about the key avoidable cancer risk factors and the actions they can take to reduce their risks. The ECAC was first published in 1987 and last updated by IARC in 2014 [5]. IARC also provided the scientific coordination for the 5th edition of ECAC (ECAC5) [30], under the World Code Against Cancer Framework [31], *that is* based on the synthesis and evaluation of the evidence on lifestyle, environmental, occupational, and infectious cancer risk factors and of effective medical interventions. The latest 4^th^ edition of the ECAC consists of a list of 12 recommendations to citizens for actions to prevent cancer [5]. While this has been evaluated for its comprehensibility, acceptability and impact on attitudes towards cancer prevention messages [32, 33], its effectiveness in increasing awareness of the risk factors for cancer, either in aggregate or in different socioeconomic groups, has not been assessed.

While all previous editions of the ECAC have relied on the same communication strategy, that is presentation of a list of recommendations to citizens for actions to prevent cancer, there are various possible ways of presenting information to optimise awareness of the risk factors and preventive actions for cancer in all socioeconomic groups. Based on an extensive literature on risk communication, some of the key factors that have the potential to influence awareness and recall of risks are message content, length and format.

### 1.1 Content—cancer prevention messages vs cancer risk messages

Framing messages in terms of the desirable consequences (gains) associated with engaging in a target behaviour or the undesirable consequences (losses) of not engaging with the target behaviour has the potential to influence attitudes, intentions and behaviours [e.g. 34]. The 4th version of the ECAC comprises a list of actions for preventing the most common avoidable cancer risk factors, *that is* framed in terms of the gains of adopting protective behaviours or stopping harmful behaviours. It is unknown whether including the risk factors for cancer, *that is* also highlighting the losses, would increase awareness of the cancer risk factors across all socioeconomic groups.

Considerable research has assessed the relative impact of gain‐framed and loss‐framed messages [e.g. 35, 36, 37, 38, 39], but the overall picture remains uncertain. Prospect theory [40], which describes how people make decisions between alternatives that involve risk, suggests that people are more sensitive to losses than they are to gains, a bias called loss aversion. Negatively framed messages tend to be more effective than positively framed ones [e.g. 41, 42, 43]. However, in the context of health communication that focuses on behaviours to prevent disease, meta‐analyses suggest that gain‐framed messages are more effective in engaging people in the content of messages and in promoting behaviour change [35, 36, 38].

In terms of messages relating to cancer prevention specifically, meta‐analytic evidence found no difference in the two ways of framing messages on attitudes or intentions to engage in either cancer prevention or cancer detection behaviours [34, 44]. Loss‐framed messages, however, were more likely than gain‐framed messages to encourage people to engage in cancer screening behaviours [34]. With regard to information recall, an unpublished study found that infographics displaying risk information for colorectal cancer were more effective than those displaying information to prevent the risks [45]. It is important to note that none of the above meta‐analyses assessed the potential for differential impact of loss‐framed *vs* gain‐framed messages in different socioeconomic groups.

The above summary highlights the uncertainties surrounding the most effective and equitable frame for conveying information about avoidable cancer risks by stopping harmful behaviours (such as quitting smoking or reducing alcohol consumption) and engaging in preventive behaviours (such as eating a healthier diet or attending screening).

### 1.2 Length of cancer prevention messages

With regard to messages about cancer, the potential impact of message length is arguably most pertinent to cancer prevention messages, rather than messages about the risk factors, given all the possible recommendations for reducing cancer risk and the level of detail that these could entail. It is important, therefore, to assess the impact of message length in relation to cancer prevention messages on awareness of cancer risks.

Brief messages are more likely to be recalled [46] and lead to greater intentions to change the target behaviour [47]. Consistent with this, messages that present fewer data and information increase awareness and speed of processing [48]. Shorter messages require less mental effort and cognitive resources to read and process and therefore have the potential to be especially effective for those with lower levels of education and health literacy—defined as ‘the ability to gain access to, understand and use information in ways which promote and maintain good health’ [49]—more often found in lower socioeconomic groups [50, 51, 52, 53, 54, 55]. On the contrary, shorter messages may involve a loss of detail, which might result in confusion, making them harder to process [56, 57]. Longer messages might better reflect the essential elements of effective risk prevention messages, rendering them more likely to persuade people to take self‐protective action [58].

### 1.3 Format

Formats requiring visual processing, such as images and infographics, have the potential to communicate information faster and more effectively than text alone [59]. The 4^th^ edition of ECAC is in text format only, without the inclusion of images.

Images and infographics may increase message appeal, resulting in greater elaboration of messages [60] and making it easier to navigate complex concepts [61]. They also have the potential to decrease cognitive load and mental effort [62] compared with the use of text. In turn, images and infographics can increase awareness and information recall [63, 64, 65, 66, 67], including of information on cancer symptoms and risk factors [68, 69]. By requiring fewer cognitive resources, visual communications can be especially effective for those with lower levels of education and health literacy.

In some studies, however, providing infographics had detrimental effects on risk perception and awareness, especially among the less health literate [70, 71, 72]. Although such findings might have been due to design features of the evaluated infographics, such as a lack of sufficient elaborating text to accompany the images, these findings highlight the uncertainty around the impact of infographics across socioeconomic groups to convey information about the avoidable cancer risk factors.

The current study aimed to inform the 5^th^ edition of ECAC by identifying the most effective method of presenting information to optimise equitable awareness of avoidable cancer risks (*i.e*. overall and across socioeconomic groups) among members of the general public of European Union (EU) member states. The specific aims were to assess the impact on cancer risk recall of messages varying in content, length and format.

## 2 Materials and methods

The study was preregistered on the Open Science Framework (registration: https://osf.io/6rjvq/; protocol: https://osf.io/pfxgs).

### 2.1 Study design

Online experimental study employing a modified (incomplete) version of a 2 × 3 × 2 factorial study design, with three factors:

i Message content: Risk factors
  - Risk factors for cancer absent.
  - Risk factors for cancer present.
ii Message length: Actions
  - Short list of actions to prevent avoidable risk factors for cancer.
  - Long list of actions to prevent avoidable risk factors for cancer.
  - List of actions to prevent avoidable risk factors for cancer absent.
iii Message format: Images
  - Text‐only.
  - Text with images.

In the absence of actions and risk factors, those allocated to the message content ‘Text with images’ would see only images, which on their own would convey little meaning. Therefore, participants allocated to this group were treated as those allocated to the absence of actions and risk factors and the message content ‘Text‐only’, and together, these two groups served as the control group (i.e. Group 1). This incomplete factorial design resulted in 10 distinct intervention groups and one control group that received no messages (Table 1).

**Table 1:** Study design showing 11 groups.

		Message content risk			
		Risk factors absent		Risk factors present	
		Text‐only	Text & image	Text‐only	Text & image
**Message length**	**Actions absent**	Control (Group 1)		Group 2	Group 3
	**Actions long**	Group 4^a^	Group 5	Group 6	Group 7
	**Actions short**	Group 8	Group 9	Group 10	Group 11

a The format of messages received by Group 4 was that used for ECAC‐4.

### 2.2 Participants

Participants were 10 027 adults (aged 18+), recruited through a specialist research agency (All Global, https://www.allglobal.com/) to be nationally representative for age, sex and education of eight EU member states. Educational level was used as a proxy measure for socioeconomic status, in line with previous research [73], given its association to economic and social resources, such as income and other material and social resources [74, 75, 76].

Two member states from each subregion of the EU (as defined by the Global Cancer Observatory, https://gco.iarc.fr/en), where the research agency had participant panels and therefore from which it was feasible to recruit, were randomly selected using the random numbers technique and an online random numbers generator (https://www.random.org): Western Europe: France and Germany; Northern Europe: Sweden and Ireland; Southern Europe: Greece and Spain; and Central and Eastern Europe: Croatia and Romania. All materials were translated by the research agency into the respective languages and were checked by respective native speakers from the ECAC Working Groups.

#### 2.2.1 Sample size planning

The sample size of 10 027 participants was estimated to provide more than 95% power to be able to detect a small effect of *d* = 0.21 or higher of Risk Factors *vs*. No Risk Factors, Short *vs*. Long Actions; and Text *vs*. Text with Images. Among the assumptions for the calculations was using the standard deviation of 1.79 found in the CRUK—Cancer Research UK's Cancer Awareness Measure (CAM) February 2023 report [23] to use a realistic parameter.

### 2.3 Setting

The study took place online, on the research agency's survey platform.

### 2.4 Interventions

Four expert working groups were tasked by IARC with revising, updating and expanding the evidence‐based recommendations of the ECAC 4^th^ edition, in order to draft the contents of the 5^th^ edition of ECAC [30] (Annex S1). Each working group identified a set of actions the general public (citizens) can follow to reduce or avoid a cancer risk relating to lifestyle determinants (Working Group 1) [77, 78]; environmental and occupational determinants (Working Group 2) [79, 80, 81]; infections and related interventions (Working Group 3) [82]; and medical interventions (Working Group 4) [83, 84]. These were edited for clarity by a fifth working group, specialising in communications and health literacy. Comprehensive details of the methods used to draft the recommendations of the ECAC‐5 are published elsewhere [85].

The draft ECAC5 was presented in one of 10 ways as described below and translated from English to all other target languages (French, German, Swedish, Greek, Spanish, Romanian and Hungarian). The information in English that was presented to each of the 10 study groups is provided in the (Annex S2). Group 1 received no intervention. All messages were designed to have a reading age index at a suitably inclusive level (B1 English level).

#### 2.4.1 Message content—Risk factors

The draft ECAC5 was presented in one of two ways: as a list of avoidable risk factors for cancer or without the list of avoidable risk factors for cancer. The risk factors were drafted by Working Group 5, based on the set of actions identified and recommended by each of Working Groups 1, 2, 3 and 4.

#### 2.4.2 Message length—Prevention actions

The draft ECAC5 was presented in one of three ways: as a list of actions for preventing cancer using long text, that is as recommended by Working Groups 1–4, or a shortened version of the text, drafted by Working Group 5. The third way involved not presenting actions for preventing cancer.

#### 2.4.3 Message format—Images

The draft ECAC5 was presented as either text alone or in combination with images depicting each risk factor. Members of Working Group 5 agreed on the images that were used, which were prepared in collaboration with graphic designers.

### 2.5 Outcome measures

#### 2.5.1 Primary outcome

Free recall of risk factors for cancer, that is mentioned from memory without being prompted, assessed using one item from the Cancer Awareness Measure (CAM), a validated measure developed to assess awareness, attitudes and behaviour in relation to cancer prevention, early diagnosis and screening [86, 87]:

*What things do you think could increase a person's chance of developing cancer? Please list as many things you can think of in the boxes below*.

Answers were scored based on the number of correct avoidable risk factors that were freely recalled. Incorrect responses, that is those relating to factors for which there is no evidence of a causal link to cancer (e.g. mobile phone use or stress) were disregarded. This measure of recall was selected to avoid ceiling effects observed when measuring awareness through recognition, in which respondents are presented with a list of preselected risk factors and asked to identify those that can cause cancer (*e.g*. in 2023, smoking, was recognised by 95% of respondents as a risk factor vs 68% who spontaneously recalled it; for drinking alcohol, recognition was 69% vs 37% for free recall; for obesity recognition was 71% vs 11% for free recall [88]).

#### 2.5.2 Secondary outcomes

- Recognition of cancer risk factors, assessed through one item adapted from the CAM [86, 87]: *Which of the following, if any, do you think could increase a person's chance of developing cancer?*, followed by a list of the risk factors as identified by the Working Groups, in addition to two nonrisk factors (use of mobile phones and feeling stressed), rated as ‘*Yes, I think this could increase a person's chance of developing cancer’; ‘No, I don't think this could increase a person's chance of developing cancer’; ‘Don't know/not sure’*.
- Engagement with messages—measured by recording the time spent viewing each version of the draft ECAC5.
- Message comprehension using one item: *‘How easy was it to understand the message you just saw about cancer?’* rated on a Likert scale from 1 (*not at all easy to understand*) to 10 (*very easy to understand*).
- Message acceptability using one item: *‘How acceptable did you find the message you just saw about cancer?’* rated on a Likert scale from 1 (*completely unacceptable*) to 7 (*completely acceptable*).

#### 2.5.3 Other measures

Demographic and other characteristics: age (inferred from data of birth), education level [assessed by asking respondents to indicate their highest educational qualification, adapted for the qualifications offered in each country and categorised as low (less than high/secondary school degree), medium (high/secondary school degree and vocational training but less than university degree) or high (university degree and higher)] and smoking status (never, former and current).

### 2.6 Procedure

Research ethics approval was obtained from the University of Cambridge, Psychology Research Ethics Committee (reference no.: PRE.2023.131) and the IARC Ethics Committee (IEC) (Reference number: IEC 24–14).

The study was conducted online using All Global's online panels. Data were collected between May and July 2024. Participants recruited from each country were randomised in equal numbers to one of 10 intervention groups or a control group that was allocated double the number of participants. Randomisation was conducted using software embedded into the research agency's survey platform. The study was undertaken with the understanding and written consent of each participant.

Depending on group allocations, participants in 11 study groups were asked to read a draft version of the ECAC5 for the general public (citizens) differing in the content of the Code—long or short prevention message, or no prevention message with or without a risk message—and the format of the Code—text or text with images.

Participants in all groups were then asked to complete the primary and secondary outcome measures. Participants in the control group were asked to complete the outcome measures without being asked to read any information about cancer. Participants in intervention groups were not able to return to the information once they had seen the outcome measures.

Following completion of the study, participants in all groups were debriefed on the detailed study aims and were given the list of risk factors for cancer seen during completion of the recognition measure, highlighting those that are true factors and those that are not (*i.e*. stress and mobile phone use).

### 2.7 Data analysis

Participant characteristics were described using frequency and percentages for qualitative variables and mean [± standard deviation (SD)] for quantitative variables.

For the statistical analysis of the primary outcome and secondary outcome of risk factor recognition, we defined an 11‐level intervention group variable, in addition to the main three factors. This variable was coded as 1 for the control group, 2 for the first intervention group and so on up to 11 for the last intervention group. While analysis using the three main factors easily quantifies the effectiveness of each factor, the use of the 11‐level intervention group variable enables comparisons between specific intervention groups and the control groups, as well as among the intervention groups themselves. To assess whether the effect of one factor or the 11‐level intervention variable on the primary or secondary outcome varied across different conditions of the confounders (e.g. by educational level), we included interaction terms in the respective models, as described below. Similarly, we also explored interactions among the three main factors.

#### 2.7.1 Primary outcome

Two approaches were used to evaluate the primary outcome data. First, a three‐way Analysis of Variance (ANOVA) was performed to examine the effects of message length, message content and message format on the number of Risk Factors Recalled (RFRs). This model included the three factors as fixed independent variables, with the number of RFRs as the dependent variable. To handle the incomplete factorial design, a dummy level of ‘no message’ was created for the message format factor. Message format therefore was considered for the analysis as having three levels: no message; text‐only; and text with image. The model was adjusted for relevant confounding factors, including gender, age (18–24, 25–49, 50–64 and 65+ years old), educational level (low, medium and high) and country. Subsequently, a linear regression model was fitted to evaluate the association between the number of risk factors recalled and the above‐mentioned 11‐level intervention group variable where the control group was considered as the reference category, adjusting for the same confounding factors as in the ANOVA analysis. These two approaches that are based on the same underlying normal distribution were complementary.

#### 2.7.2 Secondary outcomes

Recognition of all 16 cancer risk factors, engagement with messages, as well as message comprehension and acceptability, were considered secondary outcomes.

To assess the impact of message length, message content and message format on the odds of recalling all 16 cancer risk factors, a logistic regression model was fitted, adjusted for gender, age, educational level and country. Additionally, we fitted a similar logistic model using the 11‐level intervention group variable (as defined for the primary outcome analysis) to facilitate comparisons between intervention and control groups. Adjusted odds ratios (ORs) along with 95% CIs were reported.

To assess the impact of message length, message content and message format on the time spent reading the messages (engagement with messages), a linear regression model was fitted, adjusted for the same potential confounders as described above. Time was log‐transformed prior to the regression analysis to obtain an approximately normal distribution of residuals and reduce skewness. We also calculated the percentage change of geometric mean of time compared with a reference category and defined by $\left(e^{\beta }-1\right)\times 100$ where β represents the regression coefficient for a specific factor.

The impact of the interventions on message comprehension and acceptability was assessed using multinomial logistic regression models adjusted for the same potential confounders as described above.

All analyses were conducted using R software with a statistical significance level set at 5%.

## 3 Results

Participant characteristics are shown in Table 2. The number of participants from each country was similar. The majority were between 25 and 49 years old (51%), with a mean age of 43.4 years (sd = 15.2) and were female (57%). Just under 16% had low levels of education, 45% had medium levels of education, and 40% had high levels of education. The number of participants in each intervention group ranged from 856 to 797 (Table 3). The control group had 1644 participants.

**Table 2:** Participant characteristics.

	Overall (*N* = 10 027)
**Age (years)**	
18–24	13.5% (1352)
25–49	51.0% (5117)
50–64	25.4% (2549)
65+	10.1% (1009)
**Sex**	
Male	42.8% (4287)
Female	57.0% (5715)
Other/Prefer not to say	0.2% (25)
**Smoking status**	
Never	52.4% (5252)
Former	12.5% (1254)
Current	35.1% (3521)
**Country**	
Croatia	12.5% (1250)
France	12.5% (1253)
Germany	12.5% (1255)
Greece	12.5% (1251)
Ireland	12.5% (1250)
Romania	12.5% (1253)
Spain	12.6% (1259)
Sweden	12.5% (1256)
**Education level**	
Low	15.7% (1570)
Medium	44.7% (4485)
High	39.6% (3972)
**Distribution of study groups**	
Control group (Group 1)	16.4% (1644)
Risk factors only (Group 2)	9.0% (906)
Risk factors with images (Group 3)	8.3% (834)
Long list actions only (Group 4)	8.5% (856)
Long list of actions with images (Group 5)	7.9% (793)
Long list of actions with risk factors (Group 6)	8.5% (852)
Long list of actions with risk factors & images (Group 7)	8.4% (842)
Short list of actions only (Group 8)	8.4% (846)
Short list of actions with images (Group 9)	8.1% (812)
Short list of actions with risk factors (Group 10)	7.9% (797)
Short list of actions with risk factors & images (Group 11)	8.4% (845)

**Table 3:** Linear regression model for the association between the intervention group and the number of risk factors recalled. Estimates are adjusted for gender, age, educational level and country.

Parameters	Adjusted number of risks factors recalled (95% CIs)	Coefficient	Lower bound	Upper bound	*P*‐value
		0.83	0.66	1.00	<0.001
**Intercept group**					
***Reference:** no actions, no risk factors, no image/text*	1.95 (1.72; 2.18)	–	–	–	–
Risk factors only	2.55 (2.31; 2.80)	0.60	0.47	0.73	<0.001
Risk factors with images	2.53 (2.29; 2.77)	0.57	0.44	0.71	<0.001
Long actions only	2.24 (2.00; 2.48)	0.29	0.15	0.42	<0.001
Long actions with images	2.35 (2.11; 2.60)	0.40	0.26	0.54	<0.001
Long actions with risk factors	2.51 (2.26; 2.75)	0.55	0.42	0.69	<0.001
Long actions with risk and images	2.54 (2.30; 2.79)	0.59	0.45	0.73	<0.001
Short actions only	2.24 (2.00; 2.49)	0.29	0.15	0.43	<0.001
Short actions with images	2.32 (2.08; 2.57)	0.37	0.23	0.51	<0.001
Short actions with risk factors	2.48 (2.24; 2.73)	0.53	0.39	0.67	<0.001
Short actions with risk and images	2.57 (2.33; 2.82)	0.62	0.48	0.76	<0.001

### 3.1 Primary outcome: Recall of avoidable risk factors for cancer

Although the mean number of risk factors recalled was relatively low, the large sample size led to a distribution of the sample mean that approximated normality.

The unadjusted mean number of avoidable risk factors recalled across all intervention groups was 2.40 [SD: 1.72; range (min‐max): 0–14]. The proportion of participants recalling each of the 16 risk factors is shown in Table S1.

Adjusted predicted numbers of risk factors recalled according to intervention group is shown in Table 3. All intervention groups recalled a significantly greater number of risk factors compared with the no message control group (linear regression, all *P*‐values < 0.001) (Table 3). The percentage increase in the predicted numbers of risk factors recalled for the intervention groups compared with the control group ranged from 14.6% to 31.7% (Table 3).

The results showed a significant effect of message content with the inclusion of risk factors in messages significantly increasing the mean number of risk factors recalled (*P*‐value<0.001, Table 4). Adding prevention messages to messages with risk factors did not improve risk factor recall (actions: 2.18, 95% CI: 1.94–2.39; risk factors with actions: 2.41, 95% CI: 2.17–2.64; risk factor alone: 2.42, 95% CI: 2.20–2.65). There was also a significant effect of message format (ANOVA, *P*‐value <0.001; Table 4), with the inclusion of either type of message format—that is texts with or without images‐ increasing the number of risk factors recalled compared with no message. The presence of images with text, however, did not have a significant effect on recall compared with the text‐only condition (text‐only: adjusted mean = 2.38; text and images: adjusted mean = 2.43; *P* = 0.11). There was also no significant effect observed of presenting prevention messages of different lengths (ANOVA, *P* = 0.94).

**Table 4:** Results of the three‐way ANOVA analysis examining the association between the three main factors and the number of risk factors recalled, adjusted for gender, age, educational level and country.

Source of variation	Sum of squares	Degree of freedom (df)	*F* value	*P*‐value
**Message length**	0.3	2	0.06	0.943
**Message content**	93.6	1	34.3	<0.001
**Message format**	75.3	2	13.8	<0.001
**Gender**	265.6	2	48.6	<0.001
**Age**	693.8	3	84.7	<0.001
**Country**	403.5	7	21.1	<0.001
**Education level**	519.6	2	95.2	<0.001
**Within groups (Error)**	27311.2	10 007		

None of the interactions examined, either between the three main factors or between each factor and the confounders, were statistically significant (all *P*‐values >0.05). In particular, the effects of the interventions on the recall of avoidable risk factors for cancer did not vary by participants' level of education (*P*‐value for interaction = 0.86) or by country (*P* = 0.44).

### 3.2 Secondary outcomes

#### 3.2.1 Recognition of cancer risk factors

The proportion of participants who recognised each factor as contributing to the development of cancer, including the two nonrisk factors, is shown in Table S1. Eleven per cent of participants correctly recognised all 16 risk factors as contributing to cancer development while also disregarding the two nonrisk factors (11.2%; *n* = 1119). The proportion of participants that recognised all 16 risk factors according to intervention group is shown in Table 5.

**Table 5:** Proportion (*n*) of participants who recognised all 16 risk factors.

Study group	Per cent (*n*)
**Total**	11% (1117)
**Control**	4.0% (72)
**Risk factors only**	10.5% (95)
**Risk factors with images**	13% (111)
**Long actions only**	11% (93)
**Long actions with images**	11% (88)
**Long actions with risk factors**	14% (118)
**Long actions with risk and images**	14% (118)
**Short actions only**	10% (88)
**Short actions with images**	9.0% (74)
**Short actions with risk factors**	16% (129)
**Short actions with risk and images**	15.5% (131)

A logistic regression model based on the three main factors revealed positive and statistically significant associations between each factor and the odds of recognising all 16 risk factors (Table 6, *P* < 0.05). The inclusion of risk factors in messages increased the odds of recognising all risk factors by approximately 1.50 times [OR = 1.49, 95% CI (1.29; 1.73)] compared with messages not including risk factors. The length of preventive messages (short vs. long) (Table 7, *P* = 0.56) or the presence of images did not have a significant effect on the odds of recognising all risk factors (Table 7, *P* = 0.66).

**Table 6:** Adjusted^a^ odds ratios (ORs) and 95% confidence intervals (95% CIs) for the intervention factors associated with the probability of recognising all 16 risk factors.

Intervention factors	OR	Lower	Upper	*P*‐value
**Prevention message length**				
Absent	1	–	–	–
Short	1.33	1.10	1.61	0.003
Long	1.28	1.06	1.54	0.011
**Risk message**				
Absent	1	–	–	–
Present	1.49	1.29	1.73	<0.001
**Message format**				
No message	1	–	–	–
Text‐only	1.88	1.36	2.58	<0.001
Text and images	1.93	1.40	2.66	<0.001

a Model is adjusted for gender, age, educational level and country.

**Table 7:** Test linear hypothesis between several conditions based on a logistic regression model for the probability of recognising all 16 risk factors. Model is adjusted for gender, age, educational level and country.

Factors	Contrast examined	*P*‐value
**Prevention message**	Short vs long	0.56
**Message format**	Text‐only vs images	0.66

When using the 11‐level intervention group variable, results also showed positive and statistically significant associations between the intervention group and the odds of recognising all 16 risk factors (Table S2, all *P* < 0.001). All intervention groups had higher odds of recognising all risk factors as compared with the control group where, for instance, participants viewing messages including a shortened version of preventative actions with risk factors were about four times more likely to recognise all risk factors compared with the control group [OR = 4.09; 95% CI (3.03; 5.53)].

None of the interactions examined were statistically significant (*P* > 0.05).

#### 3.2.2 Engagement with messages

Prevention message and message format were positively and significantly associated with the duration of time spent reading each message (Table S3). Conversely, the presence of risk factors showed no significant association with the reading time (*P* > 0.05). Presenting prevention messages increased the duration of time spent reading messages by approximately 4%, whereas the inclusion of images or text alone led to an increase of about 7%.

The length of preventive actions (short vs. long) and the presence of images had no significant effect on duration (*P* = 0.85 and *P* = 0.79, respectively). None of the interactions examined were statistically significant (*P* > 0.05).

#### 3.2.3 Message comprehension and acceptability

The majority of participants in all intervention groups rated the messages high on comprehension and acceptability, that is gave a score above the scale midpoints (Table S4). There were no statistically significant differences in message comprehension or in acceptability between the intervention groups (*P* > 0.05). There were also no statistically significant interactions between educational level and intervention group in message comprehension and acceptability (*P* > 0.05).

## 4 Discussion

As part of the development phase of the 5^th^ edition of the European Code Against Cancer (ECAC5), the current study aimed to assess the impact of different methods of presenting information about cancer on public awareness of the avoidable risk factors for cancer. Including the risk factor alongside information about the action to prevent cancer increased by a small amount the low level of awareness of cancer risk factors in all socioeconomic groups. There was no significant effect on recall of presenting a shortened compared with a longer version of the actions, nor of including images in the messages. A similar pattern of findings was observed for recognition of cancer risks. Inclusion of information on preventative actions increased engagement with messages, but message length did not have an effect, nor did the inclusion of risk factors or images. Messages were rated high on comprehension and acceptability by all socioeconomic groups with no differences between message types.

Previous editions of the ECAC involved sets of recommendations for citizens for reducing their cancer risk. Such messages might be important for ensuring citizens have the appropriate information regarding the preventative actions they can take and while information about cancer risks might be implicit in such messages, they appear to do little to increase awareness of the main avoidable risk factors for cancer. This is important, given that a large proportion of the population lacks this knowledge [24, 25, 26]. Messages including explicit information about cancer risks were found in this study to slightly but significantly increase this awareness in all socioeconomic groups. There are three possible explanations for this finding. The first is that direct information about the risks does not require inferences to be made and is therefore more accessible and can more easily be recalled. For example, being told that alcohol is a risk factor for cancer arguably provides more direct communication about what the risk is and therefore might be easier to process and recall than being told to ‘avoid drinking alcoholic beverages as much as possible’. Indeed, direct information is generally easier to remember compared with implied information, which may require more cognitive effort to retrieve due to the need to be interpreted first [e.g. 89]. The second explanation is that cancer risk factors were presented as a simple, concise, descriptive list. When presented along with information on the preventative actions, each risk factor was listed first, with the relevant action following in a subsequent, separate line, that is the risk factor served as a header. Consistent with previous research on the effects of lists and headers on information recall and retrieval [e.g. 90, 91, 92], this may have enabled the accessibility of the information and therefore increased risk factor recall and recognition. The third explanation for the current findings is that loss‐framed messages might be more effective in conveying risk information than gain‐framed messages. Messages that include information about cancer risk factors can be viewed as highlighting losses and therefore negatively framed, while those that list preventative actions to reduce cancer risk can be viewed as highlighting the gains of adopting protective behaviours or stopping harmful behaviours. This possible explanation is consistent with research showing that loss‐framed messages are more effective than gain‐framed messages in encouraging engagement in cancer screening behaviours [34] and in increasing information recall on colorectal cancer risks [45].

The current study did not find any differences in risk factor recall or recognition of presenting cancer preventative messages of different lengths. This is contrary to findings showing that shorter messages are more likely to be recalled [46] and increase knowledge and processing speed [48], especially among those with low levels of education and health literacy [50, 51, 52, 53, 54, 55]. With longer versions in the current study, including 368 words compared with 144 words in the shorter versions, one possible explanation for this lack of difference is that perhaps the difference in length was not big enough to have an impact. Regardless of message length, however, the inclusion of cancer preventive actions increased engagement with the messages, as measured by the time spent viewing them. Although this did not result in higher recall or recognition of cancer risk factors, it is not known whether it had some other effect not measured in the current study, for example on awareness of possible cancer protective actions. Both this and awareness of the risk factors for cancer are arguably necessary for encouraging attempts to reduce cancer risk. Future research might consider complementing measures of cancer risk awareness with measures of awareness of the possible cancer preventative actions citizens can take to reduce their cancer risk.

The inclusion of images in messages also did not have an impact on recall or recognition of cancer risks in this study. This is contrary to findings showing that images and infographics can increase awareness and information recall [63, 64, 65, 66, 67], including of information on cancer symptoms and risk factors [68, 69]. We cannot exclude the possibility, however, that this was the result of the design of the images included in this study, which were arguably crude, lacking in elaborate details and small in size. Nonetheless, such images might be helpful for citizens with levels of digital and health literacy below those required to participate in an online study, such as the current one, a possibility that could be examined in future ‘offline’ research. This is important given that low levels of health literacy are associated with poorer overall health [93, 94] and, among cancer patients, poorer health outcomes, lower screening rates and low adherence to cancer treatment [95, 96, 97]. The role of more elaborate images in cancer risk awareness should also be examined in future research.

One aim of the current study was to identify the draft version of ECAC5 that could increase cancer risk awareness across all socioeconomic groups. This is especially important given that low levels of awareness are particularly marked in those in the most socioeconomically deprived groups [23]. These groups have lower levels of education and health literacy and might therefore struggle with health information. The effects observed in this study did not differ according to level of education and all messages were rated high on both comprehension and acceptability across all socioeconomic groups. This implies that a version of ECAC5 based on the current findings would be well understood and considered acceptable across all socioeconomic groups and could be equally effective in increasing awareness of the avoidable risk factors for cancer.

This study was the first attempt to evaluate and improve the impact of the ECAC, to ensure optimal and equitable cancer risk awareness in all socioeconomic groups. Its strength lies in its large sample, recruited from multiple EU countries and its randomised design, which resulted in a robust assessment of different message forms. The use of free recall to measure cancer risk awareness ensured the study tapped into citizens' actual awareness level and avoided the ceiling effects often observed with measures of recognition. The study also has a number of limitations, the most significant being related to a possible sampling bias that limits the generalisability of the findings. Participants were self‐selected and consisted of those with digital access and related digital literacy. Consequently, individuals with low levels of education and from the highest age group (65+) were underrepresented. Access to such groups might require offline, more traditional survey methods, which would complement the findings of the current online study. Furthermore, socioeconomic status was assessed only using education. Further research should consider additional relevant proxies, such as income and social status. A further limitation is that awareness of cancer risks was only assessed immediately after presentation of the draft ECAC‐5 versions. Conclusions cannot therefore be drawn regarding long‐term awareness, how this might fluctuate over time and which individual and social factors might influence it. These outcomes were beyond the scope of the current study. Also, the extent to which the slight increase in immediate awareness of the cancer risk factors observed in this study could lead to any behaviour change awaits empirical examination. Finally, it is important to note that even with presentation of the most effective drafts of the ECAC5, the mean number of risk factors recalled was low. This highlights the complexity of awareness of cancer risks, which is likely influenced by many individual and contextual factors [19, 20, 21, 22]. Increasing this awareness further will therefore likely require several interventions targeting multiple factors, beyond just the provision of information, dissemination of which will require multiple stakeholders, to target communication source references which might vary between individuals and populations.

## 5 Conclusion

**Fig. 1:**
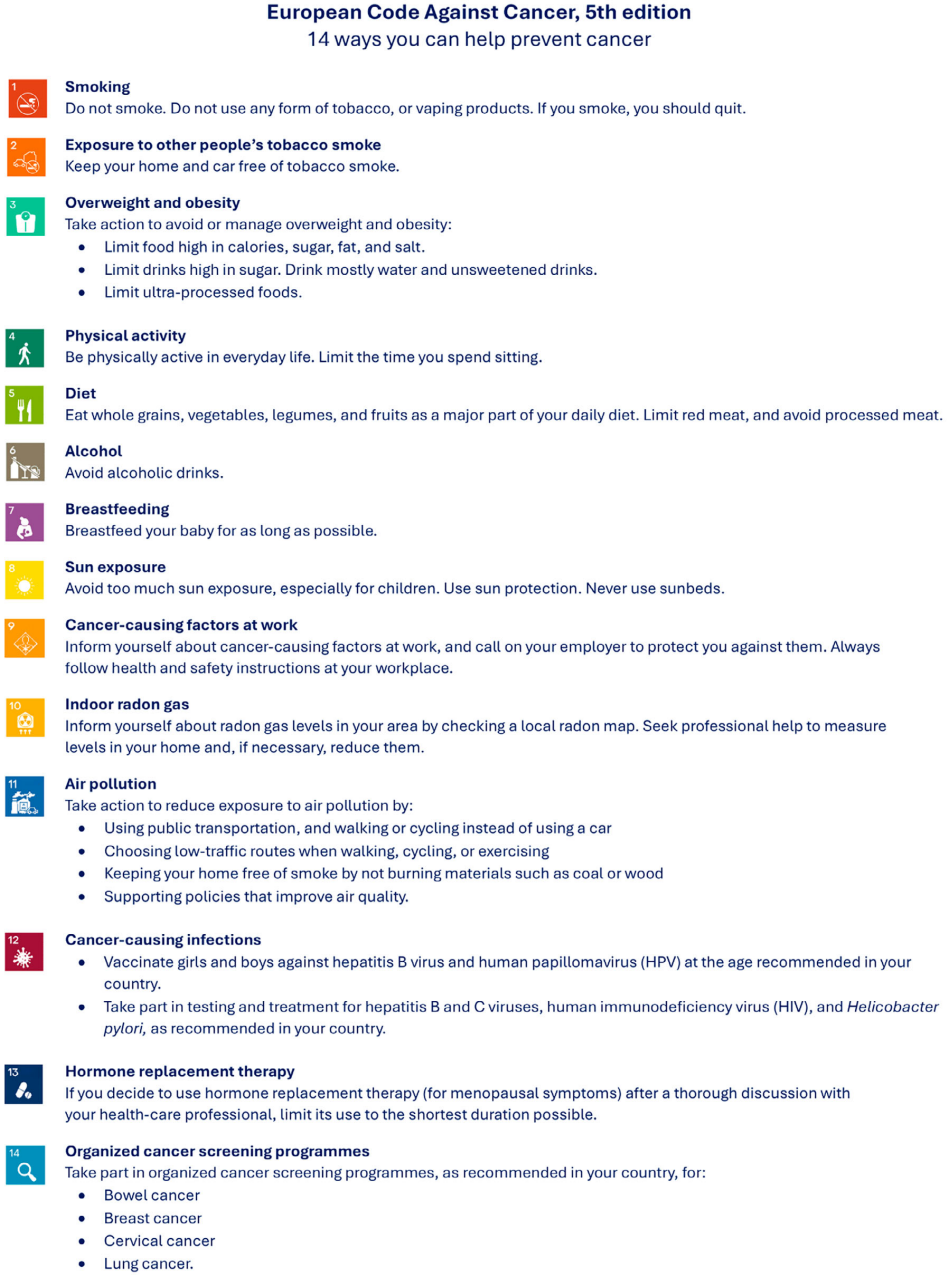
European Code Against Cancer, 5th edition: recommendations for individuals. The 14 recommendations of the European Code Against Cancer, 5th edition (ECAC5) adopted by the Scientific Committee of the ECAC5 project. © 2026 International Agency for Research on Cancer / WHO. Used with permission.

The main finding of the study was that including explicit information about the avoidable risk factors for cancer in the *European Code Against Cancer* increases the very low awareness of cancer risks among EU citizens, at least by a small amount in the short term immediately after presentation of the Code. Accordingly, the 5^th^ edition of the ECAC includes additional information on the avoidable risk factors for cancer, as well as the actions citizens can take to lower their cancer risks (Fig. 1; Annex S1). Although message length and format had no effect, longer messages about actions to prevent cancer, as well as images, provide more information to citizens without detriment. We therefore recommend considering their inclusion in dissemination campaigns of the ECAC5. In all, our recommended public communication strategy should be complemented by a collaborative approach to dissemination by multiple stakeholders, to maximise the impact of the ECAC5. The impact of the ECAC5 on health behaviour change should be the focus of future evaluations.

## Conflict of interest

The authors declare no conflict of interest. Where authors are identified as personnel of the International Agency for Research on Cancer/World Health Organization, the authors alone are responsible for the views expressed in this article and they do not necessarily represent the decisions, policy or views of the International Agency for Research on Cancer /World Health Organization.

## Author contributions

EM and TM designed the study with input from CE and DR, designed the methods with input from all other authors, collected the data and drafted the manuscript. KB advised on study design and measures. EDS designed the materials viewed by participants in intervention groups. LB analysed the data and drafted the Results section. CE, DR, KMHHB, EDS, JB, AF, CF, DP, JS, VR and HZ commented on the study protocol, findings and drafts of the manuscript.

## Supporting information


**Annex S1.** European Code Against Cancer, 5th edition. © 2026 International Agency for Research on Cancer / WHO. Used with permission.

**Annex S2.** Information presented to each of the 10 intervention groups.
**Table S1.** Proportion of participants (a) recalling unprompted and (b) recognising each of the 16 risk factors for cancer.
**Table S2.** Adjusted* Odds Ratios (ORs) and 95% Confidence intervals (95% CIs) for the group intervention associated with the probability of recognizing all 16 risk factors.
**Table S3.** Association between (log‐) time spend reading message (in minutes) and intervention factors. Estimates are adjusted for gender, age, educational level and country in multivariable linear regression.
**Table S4.** Proportion of participants scoring above messages above midpoint for comprehension and acceptability according to intervention group.

## Data Availability

The dataset generated and analysed during the current study is available on the Open Science Framework at https://osf.io/6rjvq/files/wbp5d.
