# Supplementary material for: Optimising the European Code Against Cancer, 5th edition, to increase awareness of avoidable cancer risks in all socioeconomic groups
Source: Mol Oncol. 2026 Jan 16;20(1):154–69. doi: 10.1002/1878-0261.70196 (PMC12809478; doi:10.1002/1878-0261.70196)
Supplement: Supplementary file 2 — Annex S2. Information presented to each of the 10 intervention groups. Table S1. Proportion of participants (a) recalling unprompted and (b) recognising each of the 16 risk factors for cancer. Table S2. Adjusted* Odds Ratios (ORs) and 95% Confidence intervals (95% CIs) for the group intervention associated with the probability of recognizing all 16 risk factors. Table S3. Association between (log‐) time spend reading message (in minutes) and intervention factors. Estimates are adjusted for gender, age, educational level and country in multivariable linear regression. Table S4. Proportion of participants scoring above messages above midpoint for comprehension and acceptability according to intervention group. [file MOL2-20-154-s002.docx]

**Supplement**

**Annex S1 – European Code Against Cancer, 5th edition (see separate file)**

**Annex S2 – Information presented to each of the 10 intervention groups (English)**

**Group 2 – Risk Factors for cancer only**

**
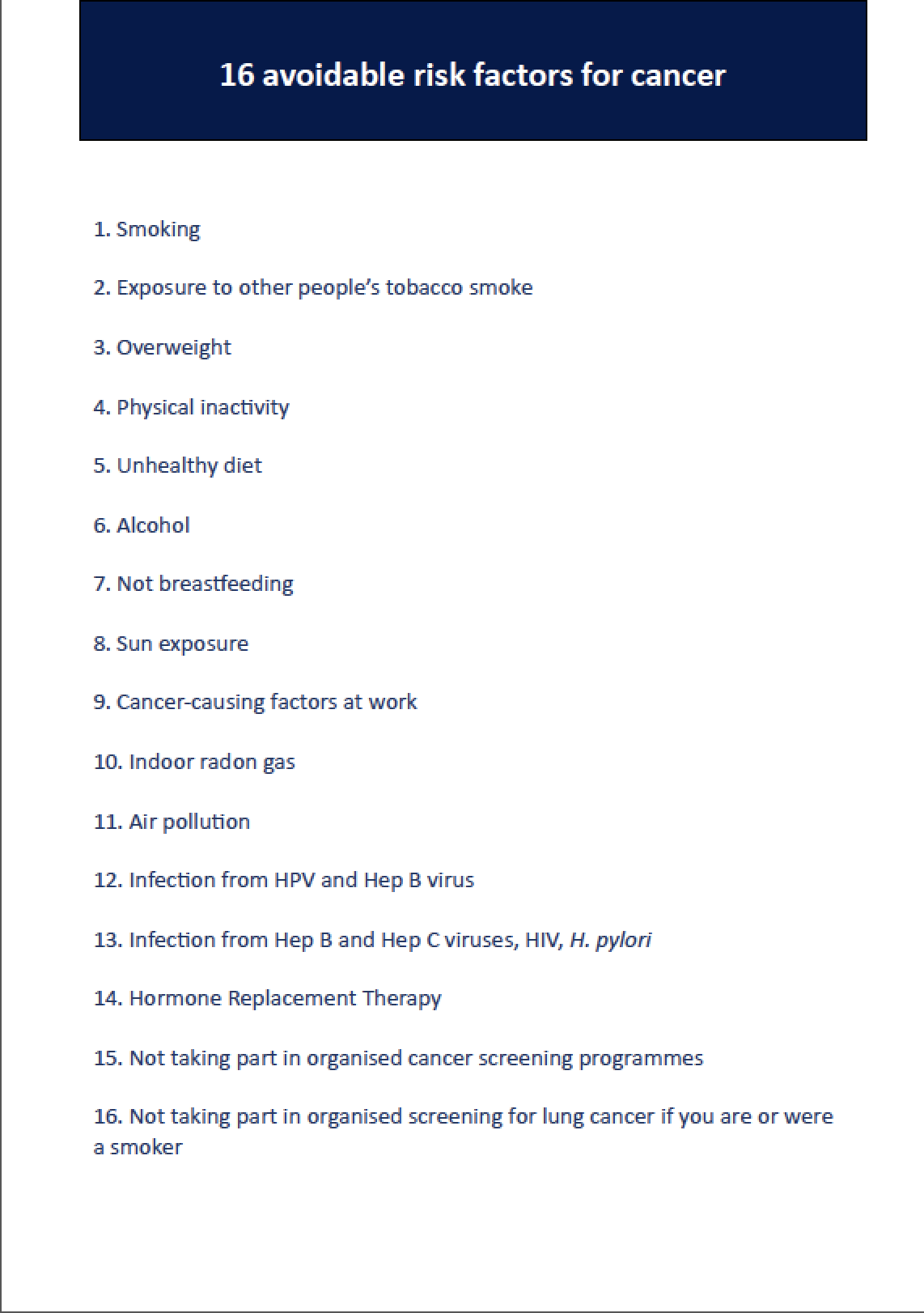
**

**Group 3 – Risk Factors for cancer with images**

**
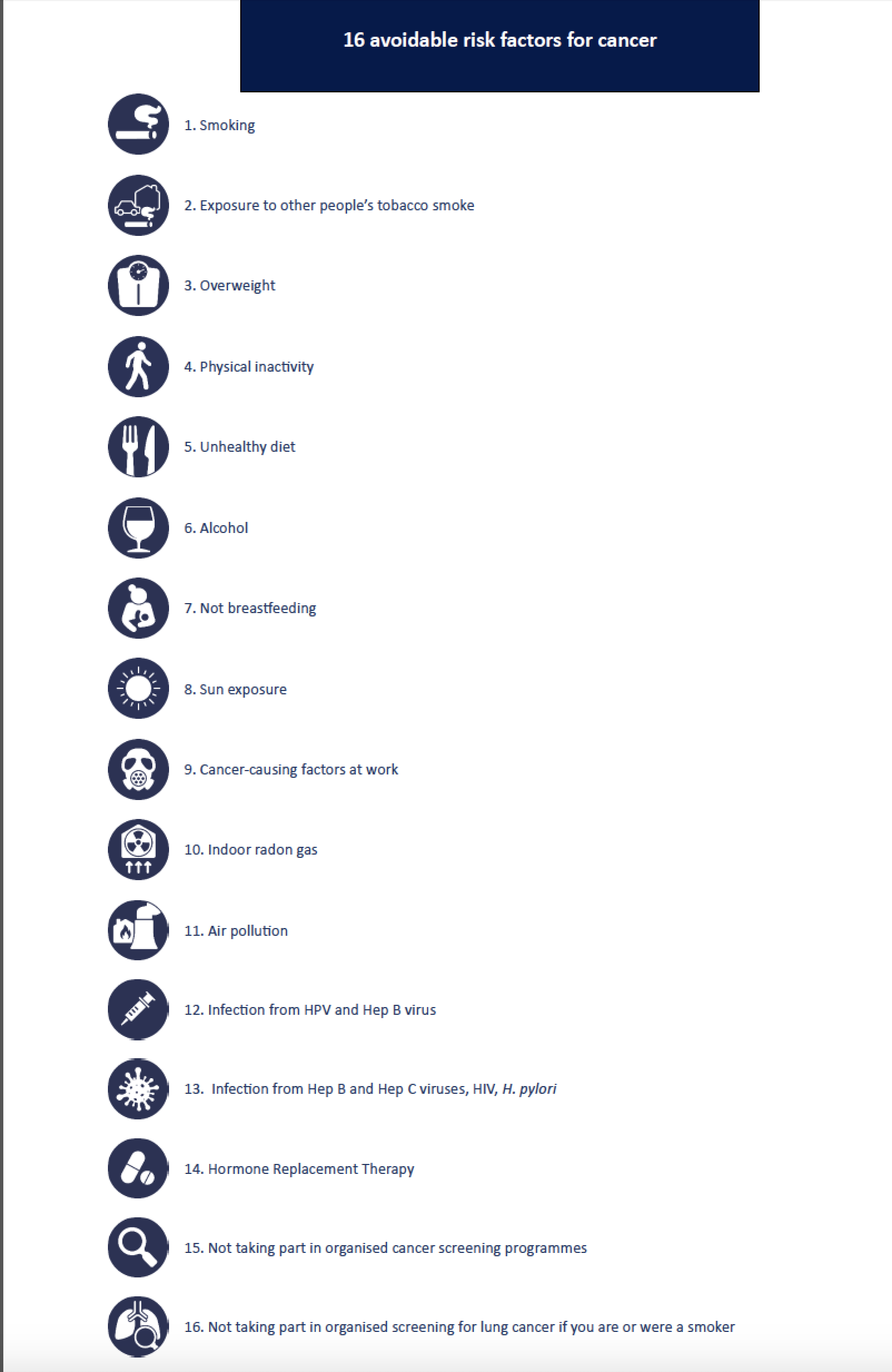
**

**Group 4 – Longer version of actions to prevent cancer**

**
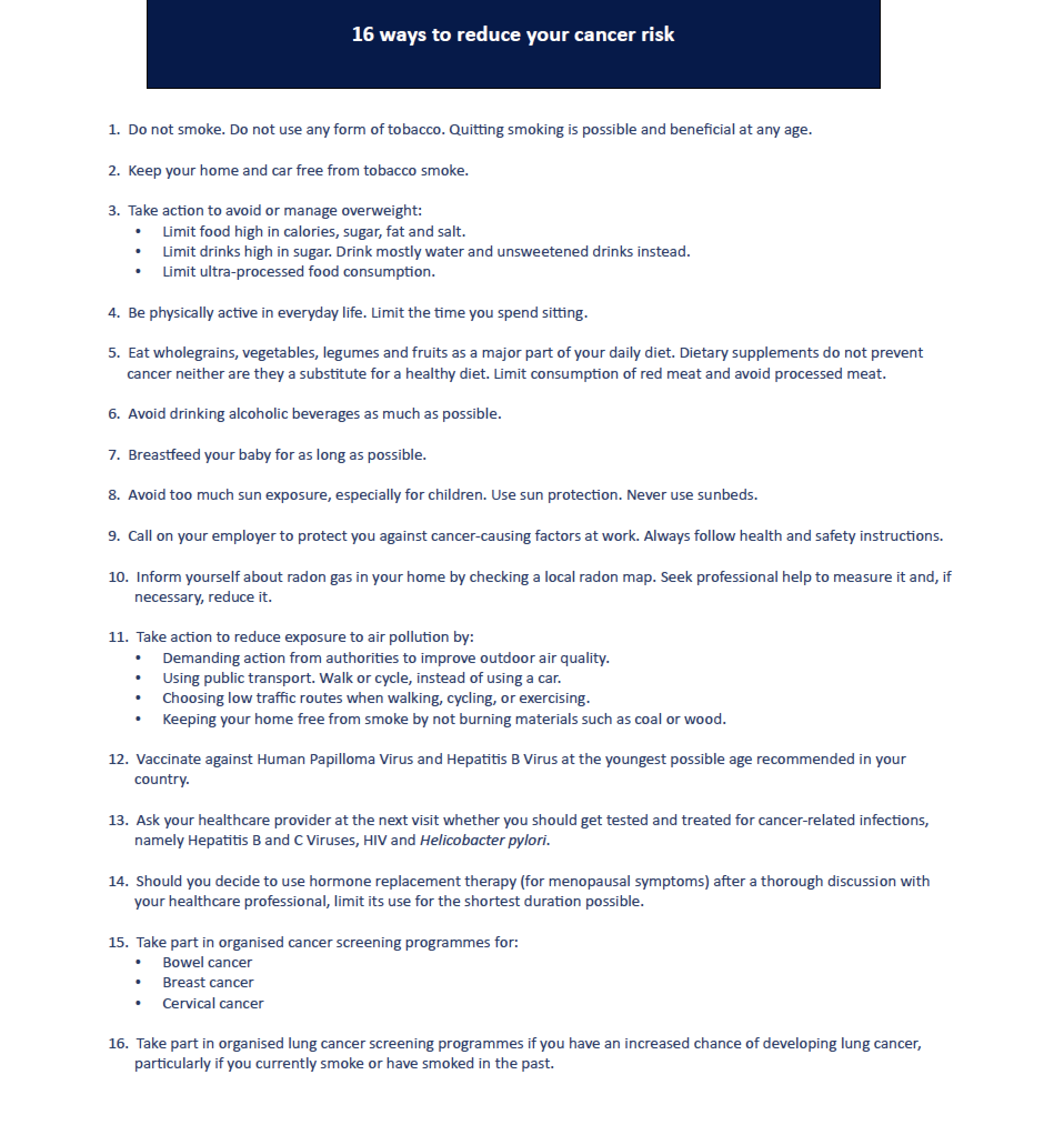
**

**Group 5 – Longer version of actions to prevent cancer with images**

**
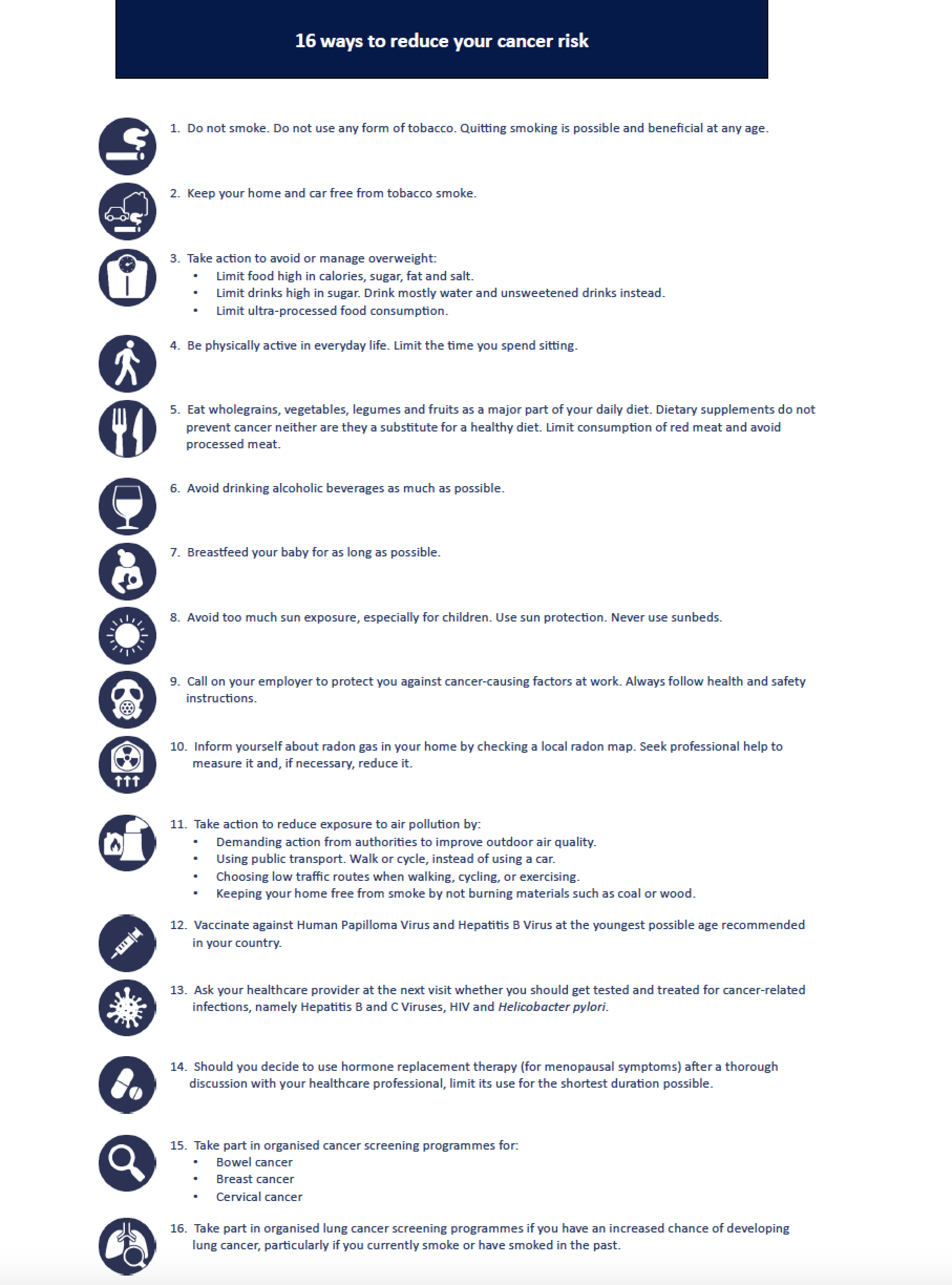
**

**Group 6 – Longer version of actions to prevent cancer with cancer risk factors**

**
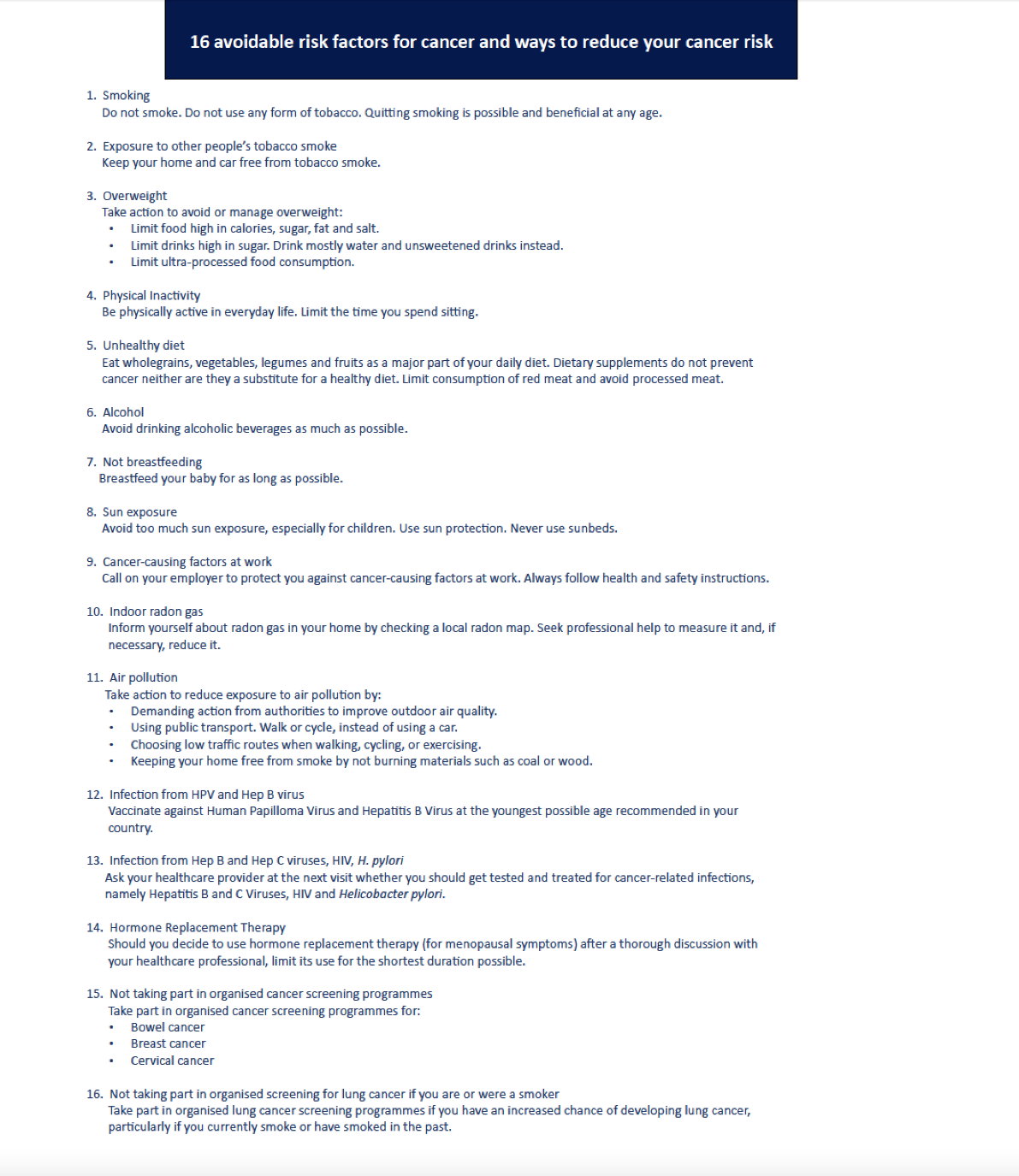
**

**Group 7 – Longer version of actions to prevent cancer with cancer risk factors with images**

**
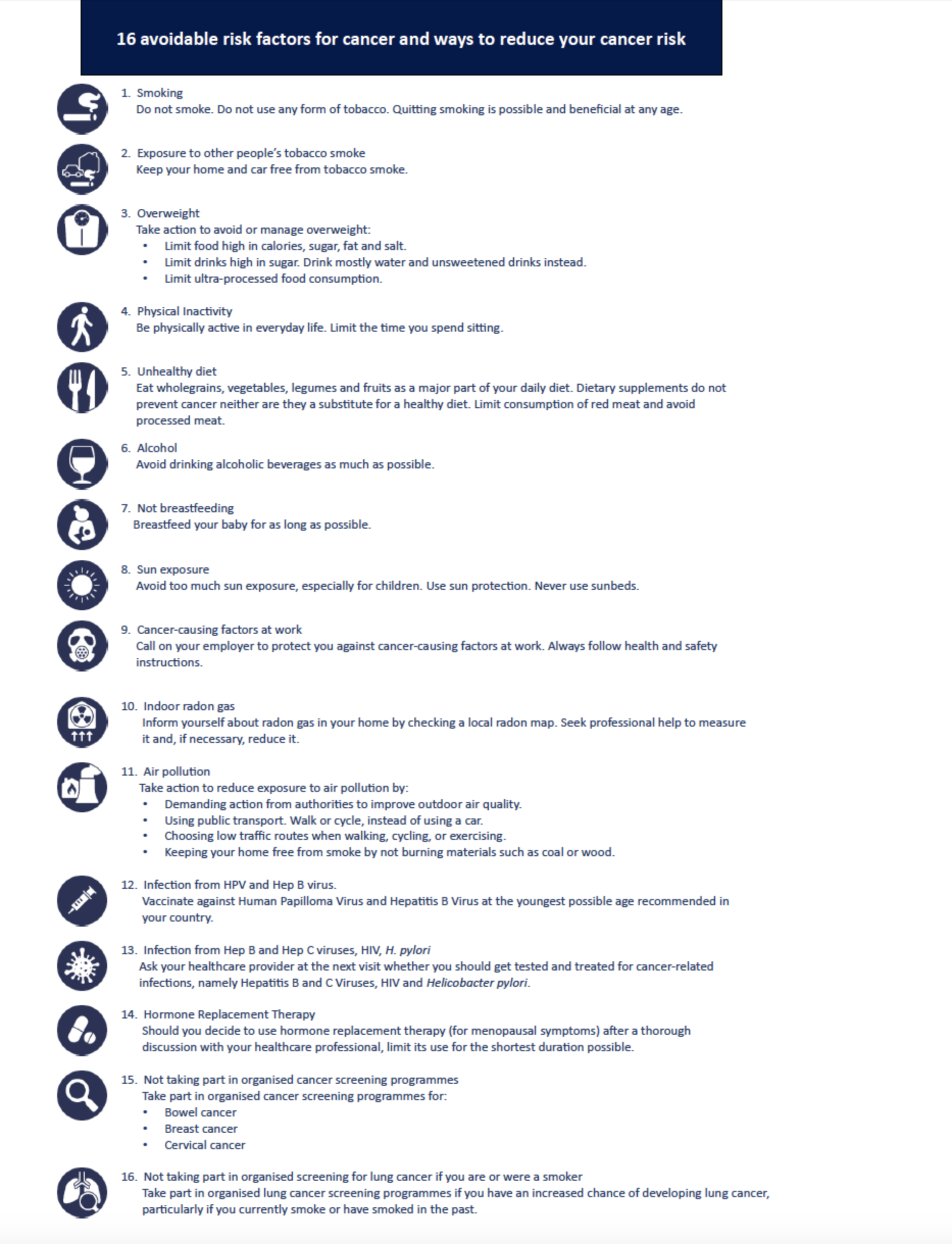
**

**Group 8 – Shorter version of actions to prevent cancer**

**
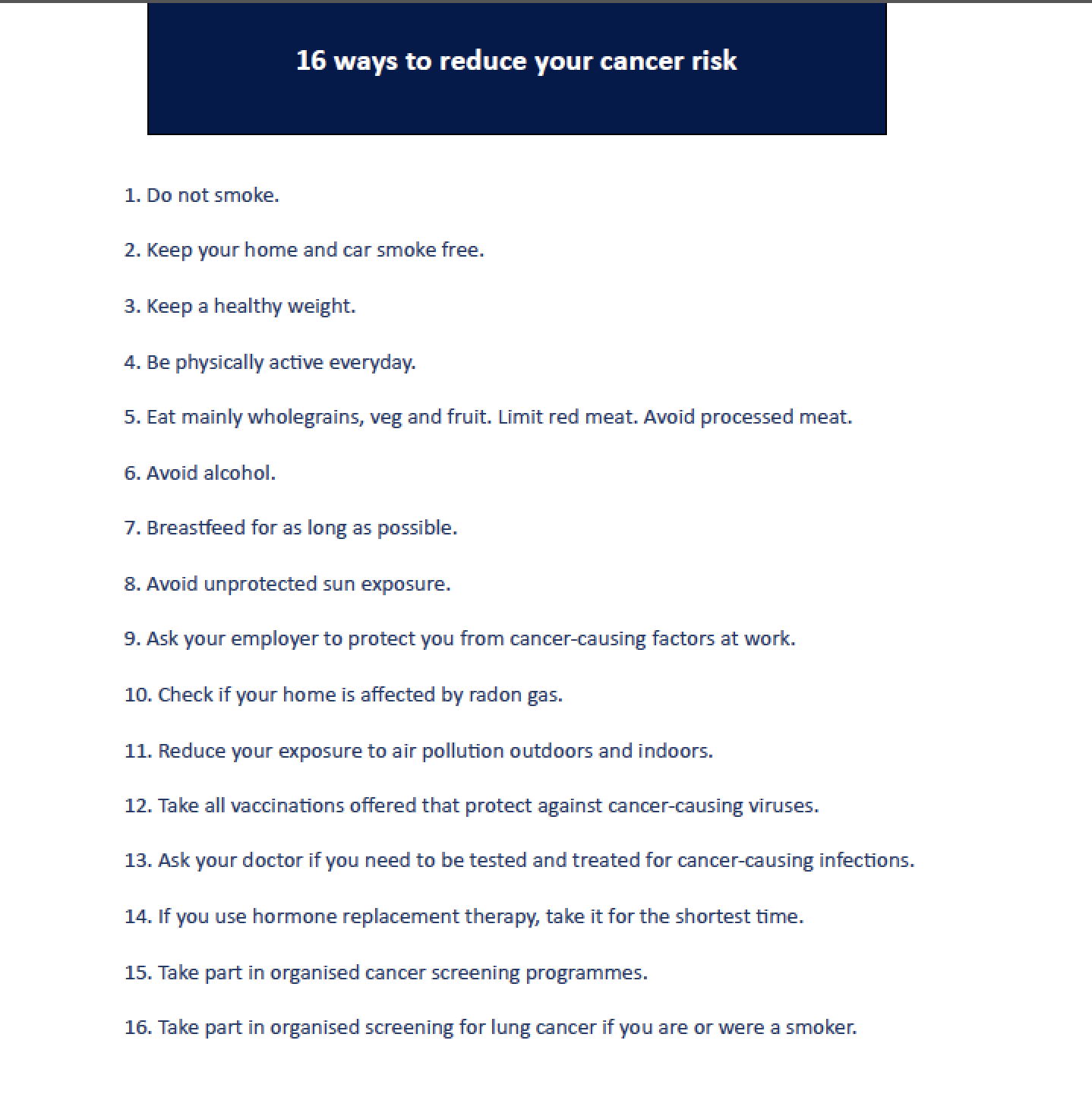
**

**Group 9 – Shorter version of actions to prevent cancer with images**

**
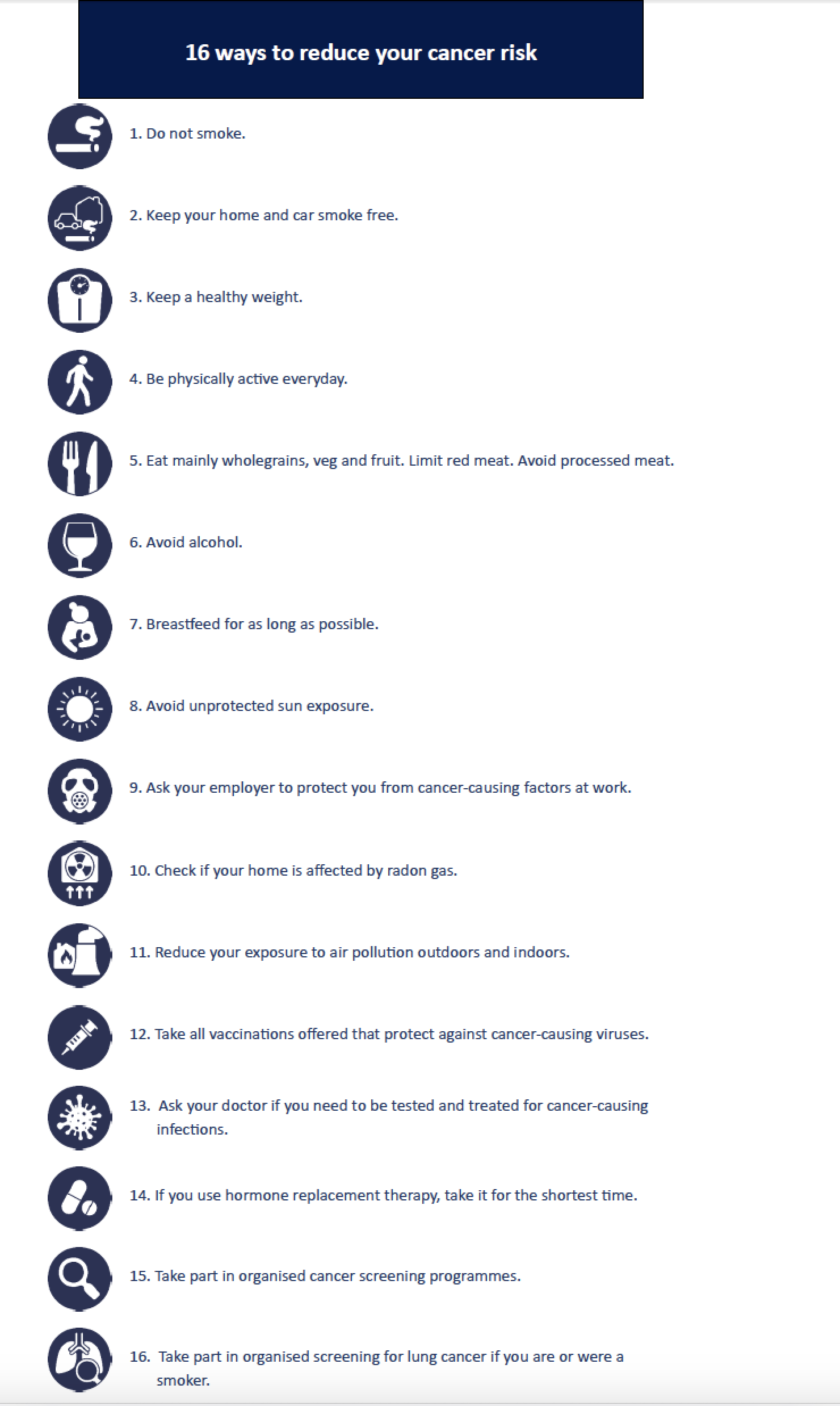
**

**Group 10 – Shorter version of actions to prevent cancer with cancer risk factors**

**
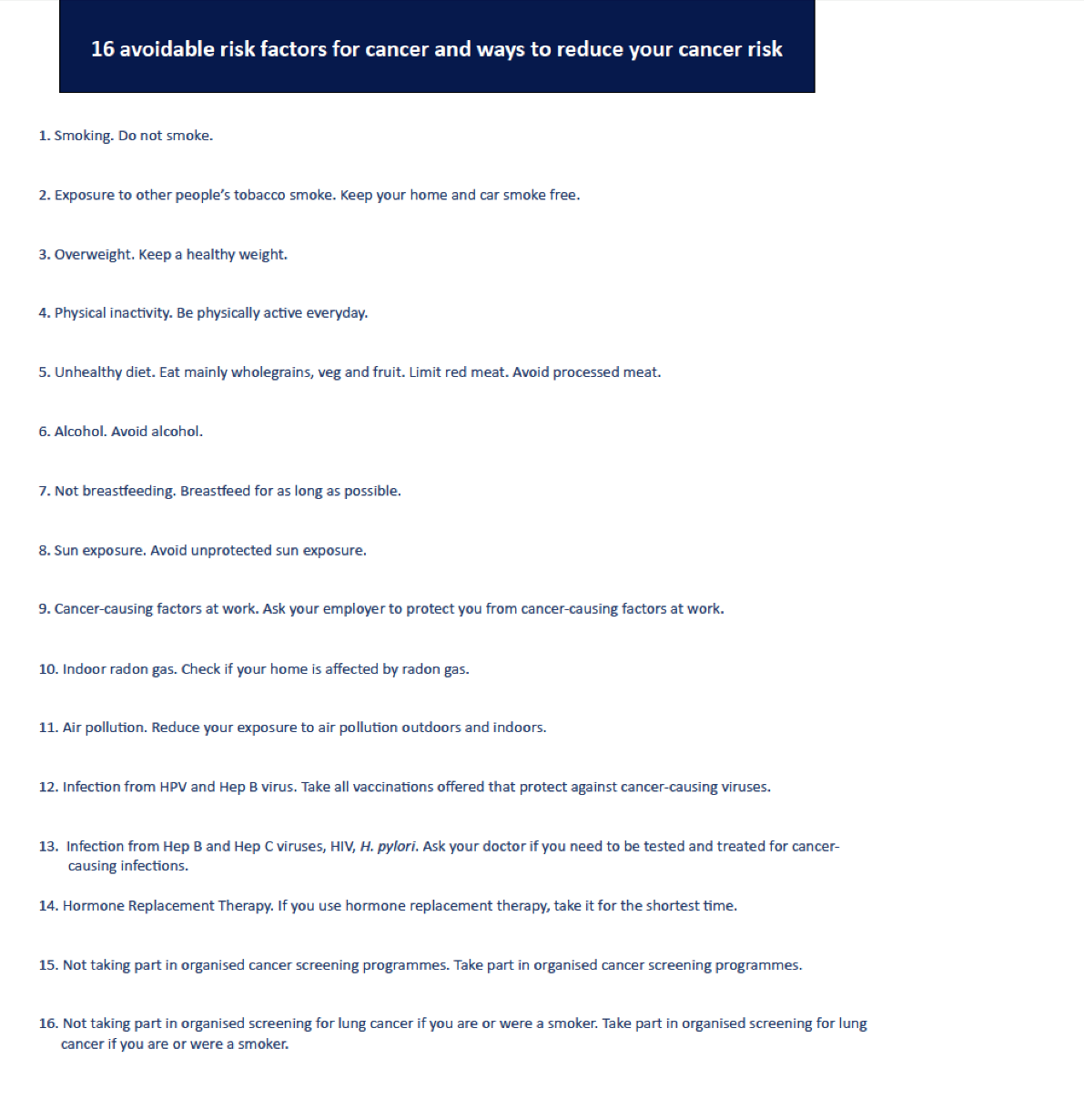
**

**Group 11 – Shorter version of actions to prevent cancer with cancer risk factors and images**

**
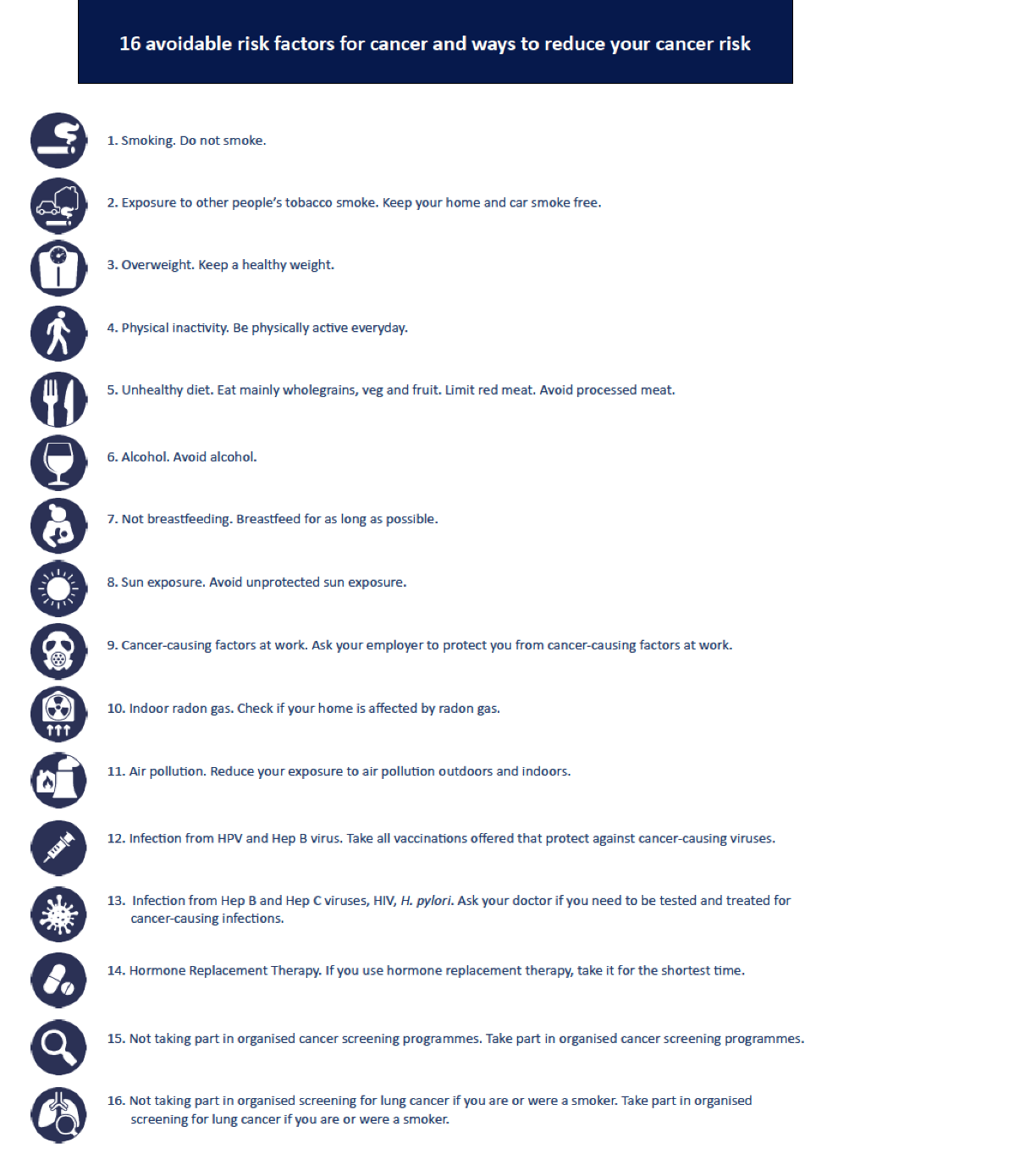
**

**Tables S1-S4**

| **Table S1:** Proportion of participants (a) recalling unprompted and (b) recognising each of the 16 risk factors for cancer | | |  | |
| --- | --- | --- | --- | --- |
| **Risk Factor** | **(a) Unprompted recall (%(n))** | **(b) Recognition (% (n))** | |  |
| Smoking | 32% (7890) | 96% (9587) | |  |
| Passive smoking | 1% (317) | 82% (8265) | |  |
| Overweight | 6% (1511) | 72% (7194) | |  |
| Physical inactivity | 7% (1754) | 65.5% (6567) | |  |
| Unhealthy diet | 20% (4977) | 85% (8491) | |  |
| Alcohol | 14% (3366) | 84% (8393) | |  |
| Not breastfeeding | 1.0% (189) | 25% (2507) | |  |
| Sun exposure | 7% (1812) | 86% (8624) | |  |
| Cancer-causing factors at work | 1% (323) | 83% (8306) | |  |
| Indoor radon gas | 1.0% (234) | 72% (7239) | |  |
| Air pollution | 5.0% (1178) | 82% (8260) | |  |
| Infection from HPV and Hepatitis B virus | 1.0% (267) | 61% (6132) | |  |
| Infection from Hepatitis B and Hepatitis C viruses, HIV, H.pylori | 1.0% (214) | 59% (5879) | |  |
| HRT | 1.0% (169) | 50% (4699) | |  |
| Not taking part in cancer screening programmes | 1.0% (281) | 52% (5212) | |  |
| Not taking part in lung cancer screening | 0% (11) | 60% (5973) | |  |

**Table S2:** Adjusted* Odds Ratios (ORs) and 95% Confidence intervals (95% CIs) for the group intervention associated with the probability of recognizing all 16 risk factors.

| **Intervention group vs controls** | **OR** | **Lower** | **Upper** | **p-value** |
| --- | --- | --- | --- | --- |
|  |  |  |  |  |
| ***Controls :*** *no actions, no risk factors, no images* | 1 | - | - | - |
| Risk Factors only | 2.49 | 1.82 | 3.42 | < 0.001 |
| Risk Factors with images | 3.22 | 2.36 | 4.38 | < 0.001 |
| Long Actions only | 2.58 | 1.88 | 3.55 | < 0.001 |
| Long Actions with Images | 2.66 | 1.93 | 3.67 | < 0.001 |
| Long Actions with Risk Factors | 3.41 | 2.51 | 4.62 | < 0.001 |
| Long Actions with Risk and Images | 3.44 | 2.54 | 4.67 | < 0.001 |
| Short Actions only | 2.48 | 1.79 | 3.42 | < 0.001 |
| Short Actions with Images | 2.22 | 1.59 | 3.10 | < 0.001 |
| Short Actions with Risk Factors | 4.09 | 3.03 | 5.53 | < 0.001 |
| Short Actions with Risk and Images | 3.90 | 2.89 | 5.26 | < 0.001 |

* Model is adjusted for gender, age, educational level, and country.

**Table S3:** Association between (log-) time spend reading message (in minutes) and intervention factors. Estimates are adjusted for gender, age, educational level and country in multivariable linear regression.

| **Intervention factors** | **Beta*** | **Lower** | **Upper** | **p-value** | **% Change** |
| --- | --- | --- | --- | --- | --- |
| **prevention message (ref = absent)** |  |  |  |  |  |
| *short* | 0.04 | 0.01 | 0.06 | 0.005 | 3.9 [1.1 ; 6.7] |
| *long* | 0.04 | 0.01 | 0.07 | 0.003 | 4.1 [1.3 ; 6.9] |
| **risk message (ref = absent)** |  |  |  |  |  |
| *present* | 0.01 | -0.01 | 0.03 | 0.194 | 1.4 [-0.70 ; 3.4] |
| **message format (ref = absent)** |  |  |  |  |  |
| *text only* | 0.07 | 0.03 | 0.10 | < 0.001 | 6.8 [3.0 ; 10.7] |
| *text and images* | 0.07 | 0.03 | 0.10 | < 0.001 | 7.0 [3.2 ; 11.0] |

* Model is adjusted for gender, age, educational level, and country.

**Table S4:** Proportion of participants scoring above messages above midpoint for comprehension and acceptability according to intervention group

| Group | Comprehension | Acceptability | |
| --- | --- | --- | --- |
| Risk factors only | 92.7% | 96.5% | |
| Risk factors with images | 91.9% | 96.0% | |
| Long list actions only | 90.7% | 95.2% | |
| Long list of actions with images | 92.3% | 94.8% | |
| Long list of actions with risk factors | 92.3% | 97.8% | |
| Long list of actions with risk factors & images | 93.0% | 96.9% | |
| Short list of actions only | 93.1% | 97.0% | |
| Short list of actions with images | 90.7% | 95.2% |  |
| Short list of actions with risk factors | 92.5%9 | 96.1% |  |
| Short list of actions with risk factors & images | 93.2% | 96.2% |  |
